# Association between sleep duration and latency, nocturnal awakenings, and body mass index among infants

**DOI:** 10.1590/1984-0462/2024/42/2023058

**Published:** 2023-12-22

**Authors:** Priscilla Márcia Bezerra de Oliveira, Márcia de Oliveira Lima, Patrícia de Menezes Marinho, Jonas Augusto Cardoso da Silveira, Risia Cristina Egito de Menezes, Giovana Longo-Silva

**Affiliations:** a Universidade Federal de Alagoas, Maceió, AL, Brazil.

**Keywords:** Body mass index, Sleep deprivation, Sleep latency, Children, Cohort studies, Índice de massa corporal, Privação de sono, Latência do sono, Crianças, Estudos de coorte

## Abstract

**Objective:**

To investigate the association between sleep duration, nocturnal awakenings, and sleep latency with body mass index (BMI) at six and 12 months of age.

**Methods:**

179 children from a birth cohort were enrolled. At six and 12 months of age, anthropometric data were obtained using standardized techniques and infants’ mothers answered the Brief Infant Sleep Questionnaire for sleep data. The association of BMI with the independent variables (sleep duration, latency, and nocturnal awakenings) was assessed by linear regression models. Analyses were adjusted for potential confounders and a p-value<0.05 was adopted to define statistical significance.

**Results:**

For each additional hour of sleep duration, BMI was reduced by 0.15 kg/m² (95% confidence interval [CI] -0.28; -0.01; p=0.03) and each additional minute of sleep latency increased BMI by 0.01 kg/m² (95%CI -0.00; 0.03; p=0.02). These associations were independent of gestational age, child sex, birth weight, duration of exclusive breastfeeding, smoking during pregnancy, and mother’s BMI, education, and marital status. Nocturnal awakenings showed no association with the outcome.

**Conclusions:**

Our findings suggest that sleep duration and sleep latency time are associated with BMI in the first year of life. Insights into the influence of sleep early in life on weight status may be helpful to complement future nutritional recommendations and prevent and treat obesity.

## RESUMO

**Objetivo:**

Investigar a associação entre duração do sono, despertares noturnos e latência do sono com o índice de massa corporal (IMC) aos seis e 12 meses de idade.

**Métodos:**

foram incluídas 179 crianças de uma coorte de nascimentos. Aos seis e 12 meses de idade, dados antropométricos foram obtidos por meio de técnicas padronizadas e as mães dos lactentes responderam ao *Brief Infant Sleep Questionnaire* para dados do sono. A associação do IMC com as variáveis independentes (duração do sono, latência e despertares noturnos) foi avaliada por modelos de regressão linear. As análises foram ajustadas para potenciais fatores de confusão e o p-valor<0,05 foi adotado para definir a significância estatística.

**Resultados:**

Para cada hora adicional de duração do sono, o IMC foi reduzido em 0,15 kg/m² (intervalo de confiança [IC]95% -0,28; -0,01; p=0,03) e cada minuto adicional no tempo de latência resultou em aumento de 0,01 kg/m² (IC95% -0,00; 0,03; p=0,02) no IMC. Essas associações foram independentes da idade gestacional, sexo da criança, peso ao nascer, duração do aleitamento materno exclusivo, tabagismo durante a gravidez e IMC, escolaridade e estado civil da mãe. Os despertares noturnos não apresentaram associação com o desfecho.

**Conclusões:**

Nossos achados sugerem que a duração e a latência do sono estão associadas ao IMC no primeiro ano de vida. Informações sobre a influência do sono no início da vida sobre o *status* do peso podem ser úteis para complementar futuras recomendações nutricionais e prevenir e tratar a obesidade.

## INTRODUCTION

Sleep is a basic biological need that acquires particular importance during early childhood due to its relationship with mechanisms of energy repair, protein synthesis, physical development and growth, and immunological and brain maturation.^1^ Thus, given the importance of the sleep component in health and the subjectivity of defining and classifying quality, the National Sleep Foundation (NSF) defined the primary indicators of sleep quality from 4 to 11 months of age: sleep duration (12–15 hours) and sleep latency (≤30 minute), determined as the duration between “turning off the lights” or getting into bed and the actual onset of sleep, and the number of nocturnal awakenings (≤1/night).^2,3^

The most frequent complaints in early childhood are excessive nocturnal awakenings and difficulties in falling asleep, present in 20 to 30% of children during the first three years of life.^4^ In addition, according to the NSF, a gradual decline in sleep duration has been observed in children around the world, causing a regular picture of sleep restriction, a finding that is corroborated by systematic reviews and multinational studies.^2,3,5,6^

This condition of sleep restriction, expressed by the short duration of sleep or insufficient daily sleep, is associated with deleterious critical aspects related to health, such as worse emotional regulation, impaired physical and cognitive growth, and excess body adiposity resulting from excessive weight gain. Therefore, the scientific community has observed a concomitant increase in the obesity epidemic and the short sleep duration in the world, especially in children.^7–9^ Furthermore, according to Morrissey et al.,^10^ in addition to duration, other quality indicators, such as sleep latency, awakenings, bedtime, and perception of sleep quality should also be considered to assess this relationship, as their investigation results demonstrate that these indicators significantly increased the odds of overweight/obesity in school-aged children.

Data evidence investigating the relationship between sleep and excess weight has been growing.^11^ Some mechanisms that justify this relationship are influenced by the deregulation of the circadian cycle caused mainly by the short sleep duration.^12^ In addition, this shortage alters the secretion and proper use of metabolic hormones, such as leptin, ghrelin, insulin, cortisol, and growth hormone, besides influencing the reduction of the basal metabolic rate.^13^

It is noteworthy that, even though childhood overweight is part of a global pandemic of complex and multifactorial etiology, which culminates in an increase in morbidity and premature mortality, sleep is configured as a promising prevention strategy in all its dimensions (sleep duration, nocturnal awakenings, and sleep latency), there are still few investigations that evaluate this relationship in children under one year of age.^14–16^

Therefore, due to the importance of sleep component in the integral health care of the child and to the lack of evidence linking very early exposure to poor sleep quality indicators and weight during the first year of life, the present study aimed to investigate the association between sleep duration, nocturnal awakenings, and sleep latency with body mass index (BMI) (mean of the 6th and 12th month) and its variation from six to 12 months of age.

## METHOD

The data used in the present study come from the birth cohort “Child Health, Food, Nutrition, and Development — SAND: a cohort study” (in Portuguese, *Saúde, Alimentação, Nutrição e Desenvolvimento Infantil*), which aimed to analyze aspects related to health, food, nutrition, and development of children from birth until the first year of life. The research was approved by the Research Ethics Committee of the Federal University of Alagoas (CAAE: 55483816.9.0000.5013), and all mothers signed an informed consent form. Methodological details are described in the investigation by Melo et al.^17^

The dyads’ enrolment occurred between February and August 2017, by consecutive sampling, in the only maternity hospital in the municipality, which is focused on low-risk pregnancies. During the 12 months of follow-up, data collection were performed in the maternity ward (at birth) and by home visits (3rd, 6th, and 12th month). In the hospital, interviews and anthropometric assessments were conducted within the first 24 hours following delivery. Follow-up home visits occurred in a 3-day window before or after the child’s birthday.^17^

Supported by a field supervisor, teams of trained registered dietitians conducted the interviews and evaluations using structured and pre-coded forms. Within 24 hours, data were entered into the database by pairs of independent operators using Epi Info™, version 3.5.4 (Centers for Disease Control and Prevention [CDC], Atlanta, USA). Eligibility criteria comprised mothers with permanent residence in Rio Largo (state of Alagoas, Brazil) and without language or speech disorders or human immunodeficiency virus/ acquired immunodeficiency syndrome (HIV/Aids) diagnosis, whose infants were born after 35 weeks of gestation and without clinical conditions that could impair normal breastfeeding initiation or food introduction.^17^

Of the total number of births (n=394) during the data collection period, 109 dyads did not meet the eligibility criteria, 41 refused to participate in the research, and one mother was identified with a language disorder, a condition that compromised communication for the interview. Therefore, there were 240 mothers and 243 children (3 twin births). At three months of age, 210 children participated in the more extensive study; at six months, 198; and at 12 months, 186 children.

**Figure 1:**
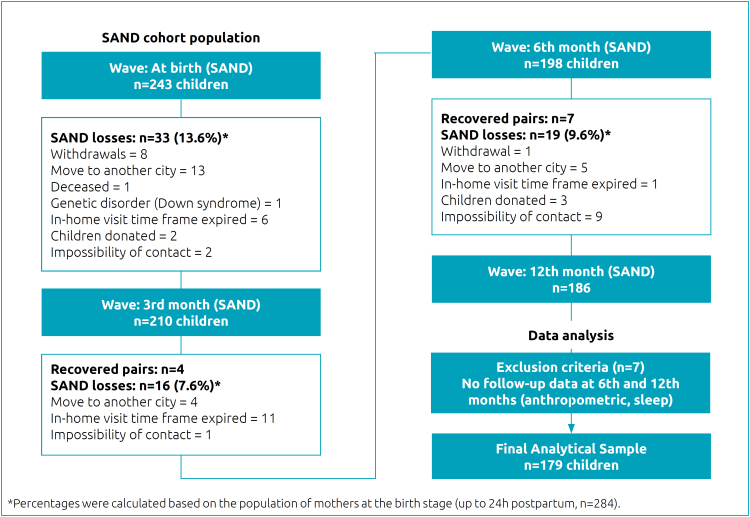
Flow diagram of the Project SAND and the selection process of the analytical sample.

Given the purpose of our study, which was to investigate sleep and BMI variables at six and 12 months, our analyses were conducted with 179 children who had complete data on these variables (Figure 1).

Due to logistic issues regarding access to dyads’ homes (i.e., absence of streets, equipment’s weight, and steep topography), it was not possible to weigh children using pediatric scales. Thus, children’s weight in household visits was calculated by the difference between the mother’s weight holding the child and her weight standing alone, using portable electronic scales P-200M (Líder; Araçatuba, SP, Brazil), with a capacity of 200 kg and 100 g precision. Recumbent length was measured using a portable infantometer (Avanutri; Três Rios, RJ, Brazil) with 1 mm precision. After positioning the child on a flat surface, two registered dietitians performed the assessment, one holding the head and the other the knees. Measurements were obtained with children without clothes and diapers and mothers dressed in light clothes and barefoot. Measurements were taken in triplicate, and we reached the final value considering the arithmetic mean.^17^

The BMI was calculated from body weight (kg) divided by the squared length (m²) in kg/m². Subsequently, the arithmetic mean of BMI kg/m² was calculated at six and 12 months. For the definition of BMI/age in Z-score, the macros of the World Health Organization were adopted for the Stata/SE 15.1 software (Stata Corp LP, College Station, TX, USA) according to child growth patterns.^17,18^

Sleep information was obtained at six and 12 months of age using the Brazilian Portuguese version of the Brief Infant Sleep Questionnaire (BISQ).^19^ This instrument allows the assessment of quality and tracking of sleep problems in children aged 0–3 years old, which investigates the typical characteristics of the child’s last week of sleep, according to the mothers’ reports. The measures used in this article were: I. Nocturnal sleep duration (from 7 pm to 7 am); II. Daytime sleep duration (from 7 am to 7 pm); III. Sleep latency (minutes); and IV. Number of nocturnal awakenings. The daily sleep duration was obtained from the sum of hours slept during the night and the day. Finally, the arithmetic means of sleep duration, sleep latency, and nocturnal awakenings at six and 12 months were calculated.

Within 24 hours after collection, data were double-entered and validated in Epi Info™ software (version 3.5.4). All analyses were performed using Stata/SE 15.1 software. For descriptive analysis, categorical and continuous variables were expressed as absolute frequencies, percentages, and means, with respective standard deviations. To assess the association between BMI (mean of 6 and 12 months), as the outcome with the independent sleep variables (6- and 12-month mean of sleep duration, sleep latency, and nocturnal awakenings) simple and multiple linear regression analyses were performed. Restricted cubic splines were also used to study the shape of the association between sleep variables and BMI.

All the multiple analyses were adjusted for potentially confounding variables: gestational age, child sex, birth weight, duration of exclusive breastfeeding, smoking during pregnancy, and mother’s BMI, education, and marital status. These variables were selected since the existence of their associations with sleep and BMI in children is present in the literature.^20–23^ A p-value (p)≤0.05 was considered statistically significant.

## RESULTS

Table 1 describes the characteristics of families, mothers, and children. The population of the present study showed a homogeneous distribution between the sexes (51.4% female). However, the prevalence of low birth weight was less than 5%, and there was a predominance of low-income families and mothers who declared themselves black or brown. As for education level, almost half of the women had less than eight years of schooling, and ∼23% were under 19 years old. Regarding breastfeeding, the exclusive offer was up to 30 days for most children (66.2%), and about ⅓ had a breastfeeding duration of fewer than 180 days.

**Table 1:** Characteristics of households, mothers, and infants. Values are expressed as number (%). (n=179).

Characteristics		n (%)
Sex*		
	Male	87 (48.6)
	Female	92 (51.4)
Birth weight (g)*		
	<2500 (low)	8 (4.5)
	≥2500 (adequate)	171 (95.5)
Skin color*		
	White	31 (17.3)
	Black or brown	147 (82.1)
	Other	1 (0.6)
Age (year)*		
	≤19	41 (22.9)
	>19	138 (77.1)
	
Education level (year)*		
	≤8	71 (39.7)
	>8	108 (60.3)
Prenatal care*		
	<6	78 (43.6)
	≥6	101 (56.4)
Per capita income (US$/day)†		
	≤5.5	101 (56.4)
	>5.5	78 (43.6)
Smoking at pregnacy*		
	No	168 (93.8)
	Yes	11 (6.2)
	
Days of exclusive breastfeeding‡		
	≤30	118 (66.3)
	>30 and ≤60	16 (9.0)
	>60 and ≤180	44 (24.7)
Days of breastfeeding‡		
	<180	56 (31.3)
	≥180	123 (68.7)

* Variables refer to birth stage (up to 24hours postpartum)

† World Bank Income Classification Criteria

‡ Variables refer to the 12-month stage;

The descriptive analysis of anthropometric variables, sleep characteristics, and their respective variations from baseline (six months) to 12 months of age is shown in Table 2. At both stages, BMI (kg/m²) means were 18.2 and 17.4, respectively. At six months, according to the BMI/age index, approximately 11% of the children were overweight, and -1.2 was the percentage variation. In addition, there was a reduction in daytime sleep duration (-2.1 hours) and an increase in nighttime sleep (0.4 hours) in the investigated period. Sleep latency means were 21.5 minutes at six months and 26.3 minutes at 12 months, representing an increase of about five minutes.

**Table 2:** Anthropometric and sleep variables of infants at six and 12 months of age and the change over six months (n=179).

		6 months	12 months	Change over 6 months
		M (SD)	M (SD)	M (SD)
Anthropometrics				
	Weight (kg)	8.0 (1.0)	9.6 (1.1)	1.6 (0.5)
	Height (cm)	66.0 (2.1)	73.8 (2.7)	7.7 (1.9)
	BMI (kg/m²)	18.2 (1.7)	17.4 (1.6)	-0.5 (1.2)
	BMI/age (Z-score)	0.7 (1.1)	0.7 (1.0)	0.0 (0.7)
Sleep				
	Sleep duration (h)	13.1 (2.43)	11.4 (2.31)	-1.6 (3.34)
	Nighttime sleep duration (h)	8.7 (1.70)	9.1 (1.86)	0.4 (2.36)
	Daytime sleep duration (h)*	4.4 (1.98)	2.3 (1.47)	-2.1 (2.51)
	Latency (min)	21.5 (16.15)	26.3 (25.94)	4.8 (25.15)
	Night awakenings (n)	1.6 (1.25)	1.4 (1.58)	-0.1 (1.45)

M: values are expressed as means; SD: standard deviation.

**Figure 2:**
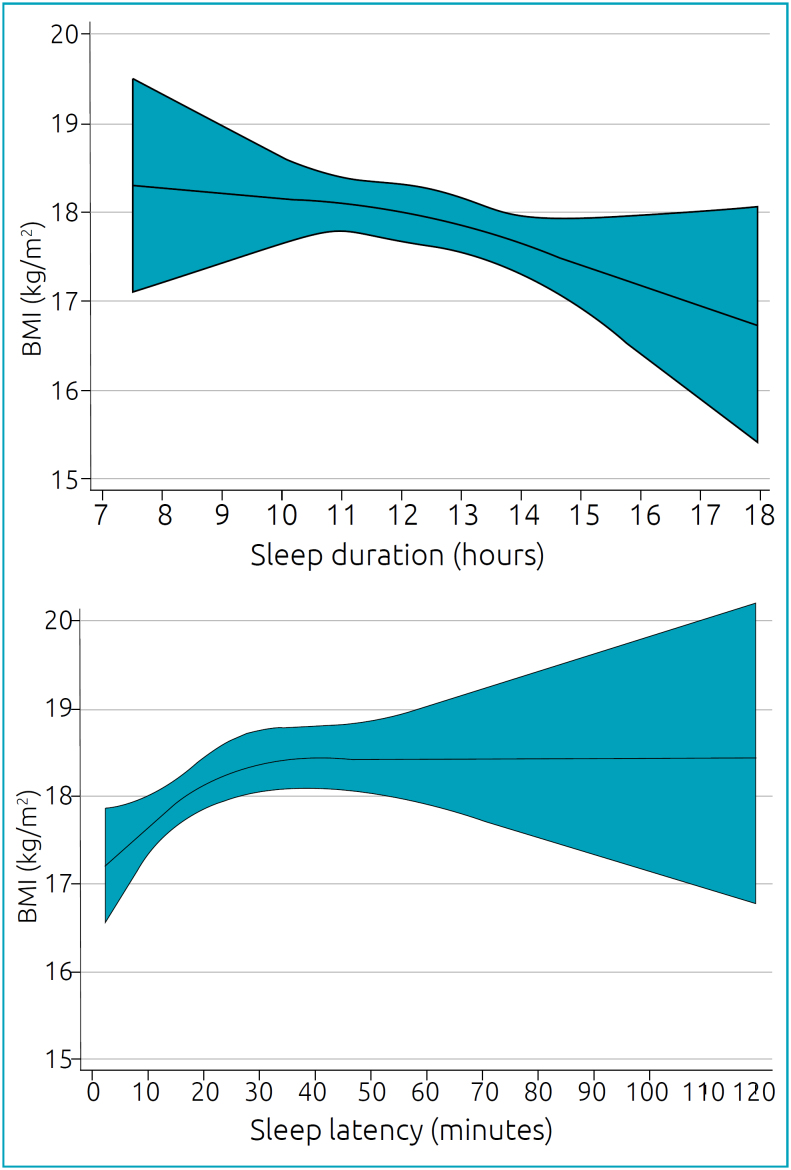
Restricted Cubic Spline Regression represents the association between mean sleep duration, latency, and BMI at 6 and 12 months. Models are adjusted for gestational age, child sex, birth weight, duration of exclusive breastfeeding, smoking during pregnancy, and mother’s BMI, education, and marital status. Black lines plot the predicted BMI values with 95%CI (grey fill).

Table 3 presents the models for the association between sleep duration, nocturnal awakenings, and sleep latency with BMI (means at six and 12 months of age). The simple regression model showed a statistically significant BMI for sleep duration (β=-0.18; 95% confidence interval [CI] -0.32, -0.47; p=0.009). When performing multiple regression analyses (adjusted for maternal and birth variables), the result practically did not change. In fact, BMI decreased by 0.15 kg/m^2^ (95%CI -0.28; -0.01; p=0.03) for each additional hour of sleep. The same multiple analysis performed with sleep latency also showed a statistically significant association, and for each additional minute of sleep latency, BMI increased by 0.01 kg/m² (95%CI 0.00; 0.03; p=0.02). Such results are also seen in Figure 2, in which restricted cubic splines modeling illustrates a dose-response association of sleep duration and latency with BMI.

**Table 3:** Associations between infants’ sleep duration, night awakenings, and sleep latency with BMI. Linear regression models were adjusted for gestational age, child sex, birth weight, duration of exclusive breastfeeding, smoking during pregnancy, and mother’s BMI, education, and marital status.

Sleep variables	BMI (kg/m²)*			
	Unadjusted model		Adjusted model	
	β (95%CI)	p-value	β (95%CI)	p-value
Sleep duration (h)	-0.18 (-0.32; -0.47)	**0.009**	-0.15 (-0.28; -0.01)	**0.030**
Night awakenings (n)	0.04 (-0.15; 0.23)	0.670	- 0.03 (-0.23; 0.16)	0.750
Latency (min)	0.01 (-0.00; 0.02)	0.050	0.01 (0.00; 0.03)	**0.020**

BMI: body mass index; CI: confidence interval.

* Mean BMI at six and 12 months. Bold values denote statistical significance at the p<0.05 level.

No significant associations were found between the number of awakenings and BMI.

## DISCUSSION

The main results were that for each additional hour of sleep, BMI was reduced by 0.15 kg/m², and each additional minute in sleep latency implied an increase of 0.01 kg/m² in BMI. These associations were independent of birth weight, maternal smoking during pregnancy, breastfeeding duration, maternal education, and child sex.

Our findings contribute to the robustness of the evidence of these relationships in a little-investigated age group in the scientific community. Findings are also strategic in order to highlight the importance of incorporating the sleep quality theme into care protocols among childcare routines, international surveys, and documents on children’s health. It is, therefore, an essential contribution to a major health problem — overweight in children, considering that the first 1,000 days of life (from conception up to two years of age) consist of a period of paramount importance regarding early prevention of obesity.^24^

Despite the scarcity of studies that investigated the relationships between sleep quality indicators and weight status among infants, our findings agree with similar data from research conducted in other age groups.^10,21,25^ For example, Chaput et al.^5^ in a cross-sectional and multinational study that aimed to analyze the relationship between lifestyle and obesity in 12 countries, showed that almost 60% of children between nine and 11 years old slept less than the recommended mean.

The increase in weight status resulting from sleep deprivation can be explained by changes in different physiological mechanisms related to body weight control, including decreased glucose tolerance and insulin sensitivity, and increased nocturnal cortisol concentrations favoring fat accumulation. Besides, reduction in growth hormone secretion and disruption of normal endocrine functioning, with a decline in leptin (appetite suppressant) and an elevation in ghrelin (appetite stimulator), are changes that directly contribute to the likelihood of developing an unfavorable BMI. Such tendencies were demonstrated in previous studies, showing that increasing sleep in young children is one of the most promising strategies for preventing childhood obesity.^25–27^

It should be added that, in most cases, sleep-related problems do not arise from intrinsic sleep disorders that require continuous medical care but rather from behavioral problems influenced by environmental factors. Some examples are excessive exposure to artificial light and screens, especially at nighttime, and parental behavior (such as not setting healthy sleep habits by establishing a bedtime routine with regular times to sleep and wake up), indicating that short-term changes in these aspects may help improve sleep quality.^16,28^

Therefore, sleep-related problems are part of the list of modifiable risk factors for excessive weight gain. Such a relationship suggests that the use of subjective tools to measure habitual practices and sleep quality in pediatric clinical practice, as well as in routine protocols in primary care, may represent an effective strategy for the early identification of risk factors for obesity and other outcomes arising from poor sleep quality in childhood growth and development. In addition, it is well elucidated in the literature that sleep and weight patterns in childhood tend to be maintained throughout life, predisposing adverse health outcomes, such as psychological morbidities, cardiovascular and joint diseases, and other conditions that impair quality of life and increase economic burdens.^21,29^

Our study has some strengths. Firstly, we emphasize that we used data from the first and only birth cohort in the state of Alagoas (Brazil). In addition, we conducted interviews at fixed times (at six and 12 months postpartum) to minimize age variability among children. Although more accurate information on sleep behavior can be obtained through objective measurements, there is evidence that the mother’s reports are consistent with actigraphy measurements. Furthermore, since the scale used in the present study (BISQ) is characterized as a psychometric tool, clinical and ecological support for clinical and research purposes are widely used in international studies.^30^ On the other hand, some limitations must be considered. Although loss minimization strategies are foreseen in cohort studies, follow-up losses still occur. More than 20% of sample loss can compromise the study’s internal validity. However, comparative analyses were not performed between children with missing and complete data to minimize this effect.^31^ Finally, the children studied represent the population of a vulnerable region of Brazil; for this reason, the interpretation of the results must be considered with caution in populations with different socioeconomic characteristics.

Therefore, our findings suggest that sleep duration and latency are associated with BMI in the first year of life. Insights into the influence of sleep early in life on weight status may be helpful to complement future nutritional recommendations and prevent and treat obesity. We should emphasize the need for further studies, mainly longitudinal and in the age group studied here, to better understand the causality and impact of sleep on long-term weight gain.
